# The Ethnopharmacological, Phytochemical, and Pharmacological Review of *Euryale ferox* Salisb.: A Chinese Medicine Food Homology

**DOI:** 10.3390/molecules28114399

**Published:** 2023-05-28

**Authors:** Jiahui Jiang, Haiyan Ou, Ruiye Chen, Huiyun Lu, Longjian Zhou, Zhiyou Yang

**Affiliations:** 1 Guangdong Provincial Key Laboratory of Aquatic Product Processing and Safety, Guangdong Province Engineering Laboratory for Marine Biological Products, Guangdong Provincial Engineering Technology Research Center of Seafood, Key Laboratory of Advanced Processing of Aquatic Product of Guangdong Higher Education Institution, College of Food Science and Technology, Guangdong Ocean University, Zhanjiang 524088, China; 2112203057@stu.gdou.edu.cn (J.J.); 2112203092@stu.gdou.edu.cn (H.O.); 2112203075@stu.gdou.edu.cn (R.C.); 2112203072@stu.gdou.edu.cn (H.L.); 2 Collaborative Innovation Centre of Seafood Deep Processing, Dalian Polytechnic University, Dalian 116034, China

**Keywords:** *Euryale ferox*, traditional medicine, phytochemical constituents, pharmacological effects

## Abstract

*Euryale ferox* Salisb. (prickly water lily) is the only extent of the genus Euryale that has been widely distributed in China, India, Korea, and Japan. The seeds of *E. ferox* (EFS) have been categorized as superior food for 2000 years in China, based on their abundant nutrients including polysaccharides, polyphenols, sesquineolignans, tocopherols, cyclic dipeptides, glucosylsterols, cerebrosides, and triterpenoids. These constituents exert multiple pharmacological effects, such as antioxidant, hypoglycemic, cardioprotective, antibacterial, anticancer, antidepression, and hepatoprotective properties. There are very few summarized reports on *E. ferox*, albeit with its high nutritional value and beneficial activities. Therefore, we collected the reported literature (since 1980), medical classics, database, and pharmacopeia of *E. ferox*, and summarized the botanical classification, traditional uses, phytochemicals, and pharmacological effects of *E. ferox*, which will provide new insights for further research and development of EFS-derived functional products.

## 1. Introduction

**Figure 1:**
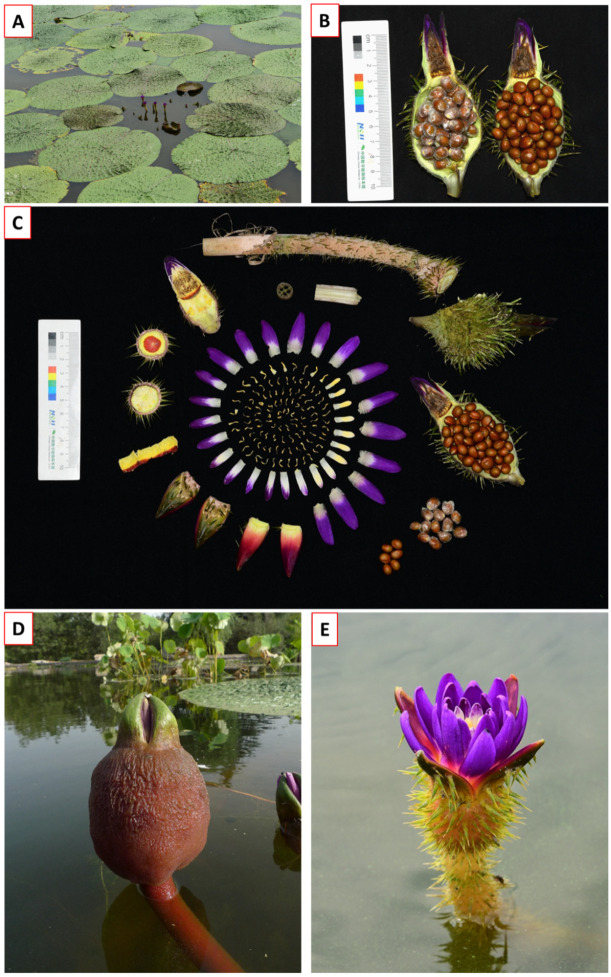
The biotope and morphological characteristic of *E. ferox*. (**A**) The leaves of *E. ferox*. (**B**) The seeds of *E. ferox*. (**C**) the pedicels, receptacles, sepals, and corolla of *E. ferox*. The fruits of Southern *E. ferox* (**D**) and northern *E. ferox* (**E**) are presented.

*Euryale ferox* seed (EFS) is a typical representative of “a medicine food homology species”, as described in the Huangdi Nei Jing Tai Su (黄帝内经太素): “Eating it as food on an empty stomach, and taking it as medicine for patients” [1,2]. EFS is the dried seeds of the *E. ferox* Salisb. plant (Figure 1A), and is widely distributed in India, Bangladesh, Myanmar, New Zealand, Russia, Thailand, and parts of East Asia [3,4]. In China, EFS was first described in “Shen Nong’s Classic of the Materia Medica” (Shén Nóng Bĕn Căo Jīng, 神农本草经) [5]. EFS, also known as Foxnut, Lotus seeds, Gorgon nuts, and Phool Makhana, is spherical, commonly broken grains (Figure 1B) [6,7,8].

As a folk medicine in China for thousands of years, EFS is primarily used to reinforce the kidney, invigorate essence, and tonify the spleen to arrest diarrhea. It is commonly employed to manage conditions such as spermatorrhea, gonorrhea, dysmenorrhea, incontinence of urine, and diarrhea of the bowels [9]. In March 2002, the National Health Care Commission of the People’s Republic of China embodied EFS as one of the new herbal medicines on the list of medicinal and food ingredients, with the stipulation that it can be used for both medicinal and food purposes within a limited range and dosage. The dried ripe seed is included in the Chinese Pharmacopoeia (2020 Edition) as a commonly used Chinese herbal medicine. According to the theory of Chinese medicine, it tastes sweet, bitter, and astringent, and attributes to the spleen and kidney meridians [9]. The raw EFS comprises about 61% carbohydrates, 12.1% moisture, 15.6% protein, 1.35% fat, 7.6% fiber, and 1.8% minerals, and possesses a calorific value of 362 kcal/100 g [10]. EFS contains an assortment of chemical constituents including triterpenoids, sterols, flavonoids, phenylpropanoids, organic acids, essential oils, and polysaccharides, while triterpenoids and flavonoids are considered major active components [8,10,11,12]. Modern pharmacological studies indicate that it exerts a wide range of bioactive activities, such as anti-tumour, anti-bacterial, anti-viral, anti-inflammatory, immunomodulatory, hypotensive, hypoglycaemic, hypolipidaemic, anti-oxidant, free radical scavenging, and hepatoprotective effects [13,14,15,16,17,18,19,20,21,22,23]. More importantly, its excellent qualities and remarkable efficacy have also been highlighted in clinical applications, which are mainly used for the treatment of cancer, hypertension, diabetes, pelvic inflammatory disease, thyroid, and prostrate disorders [24].

*E. ferox* is widely distributed throughout tropical and subtropical regions of Asia and Southeast Asia. India, Japan, Korea, Bangladesh, and China are the main producing areas [6]. Generally, *E. ferox* is grown in stagnant water with a depth of 0.2–2.0 m, such as ponds and lakes (Figure 1A). It prefers warm and sunny weather and is intolerant to cold and drought. The suitable temperature ranges from 20 to 30 °C, while fertile soil with sufficient organic matter is required [6,25]. The plant bears 8–9 spherical leaves, while bright purple flowers are arranged in alternating rows and interlaced similar to an octopus. The flower is epigynous with more than 40 corollas, ovary 7–16 chambered with 6–8 seeds/locule. The seeds are spherical with a diameter of 0.5–0.8 cm, the surface is covered with a brownish-red or reddish-brown inner seed coat, one end is yellow-white, accounting for about 1/3 of the whole, and there is concave seed cleft hilum. The roots are long, fleshy, fibrous, usually in 2–3 clusters with numerous stomata (Figure 1C) [7]. The whole plant of *E. ferox* is edible except for the leaves [6]. In China, *E. ferox* is divided into southern and northern varieties. Northern *E. ferox*, also known as prickly *E. ferox*, is a wild species with purple flowers. It is mainly distributed in Hongze Lake and Baoying Lake of Jiangsu province (Figure 1E). Southern *E. ferox*, also known as Su Qian, is a variety of northern *E. ferox* after artificial domestication and cultivation, with larger leaves and white, red, or purple flowers (Figure 1D). Nevertheless, the southern *E. ferox* is mainly used for food and export, while the northern *E. ferox* is for medicinal applications [1,4].

The National Research Center for Makhana, Darbhanga (ICAR) estimates that the total area of *E. ferox* cultivation in India is 15,000 ha, while in China, Shangrao, Jiangxi Province is the biggest cultivation city with 6700 ha every year. In India, 120,000 metric tonnes of *E. ferox* seeds were produced each year, the produce is valued at Rs 250 crore at the farmer’s end. India exports about 1–2% of its overall output and almost 100 tonnes of popped *E. ferox* are shipped each year [10]. It is worth noting that the growing and processing of *E. ferox* is laborious and demanding work. To maintain plant-to-plant spacing, thinning seedlings are required after planting, while seeds collected from the pond are needed during harvesting. Subsequently, processing including storage, cleaning, grading, heating, and tempering is required, all of which are performed manually. The whole operation is therefore strenuous and painful work from planting to harvest.

Although the main constituents and bioactivities of EFS have been extensively investigated, detailed summaries are few. Furthermore, the pharmacological mechanisms of active components remain to be reviewed. Thus, we aim to perform a comprehensive, in-depth, and systematic review of EFS’s traditional applications, phytochemistry, pharmacological effects, and toxicity over the past 30 years. This review will provide quotable evidence for future studies on EFS and contribute to shedding further light on the biological activities and clinical applications of EFS.

## 2. Methodology

To retrieve information relevant to this review, an extensive search of the literature was conducted using multiple databases, including Google Scholar, Web of Science, PubMed, SciFinder, China National Knowledge Infrastructure (CNKI), and the China Science and Technology Journal database. The main keywords searched were “*E. ferox*”, “Foxnut”, “Lotus seeds”, “Gorgon nuts”, or “Phool Makhana”. Subsequently, the keywords “chemical composition”, “phytoconstituents/phytochemicals”, “biological activity”, “pharmacological activity”, and “toxicology” were used to refine the publications. *E. ferox*-related articles were retrieved from peer-reviewed journals worldwide ranging from 1980 to 2023. These articles were read thoroughly during the compilation and integration of the information to evaluate the authenticity and relevance of their information. Studies lacking scientific names were excluded, as well as studies on the uses of EFS in fields other than the medical and nutritional sciences. All chemical structures were validated by Sci Finder and drawn using Chem Draw Ultra 15.0.

## 3. Traditional Medicinal Uses of *E. ferox*

The unique natural conditions and long-term practice in different regions have led to versatile lifestyles and unique experiences in the treatment of certain diseases. EFS is generally used as an economic crop in India, Japan, Korea, Bangladesh, and other Southeast Asian countries [26]. In China, EFS was first recorded in “Shen Nong’s Classic of the Materia Medica” and described as “sweet and astringent tastes, and used for dampness and paralysis, pain in the lumbar spine and knees, tonifying and removing malignant diseases, benefiting the essence, strengthening the will, and making the ears and eyes wise” [5], and most of the subsequent records followed this statement with modifications. Current Chinese Pharmacopoeia (2020 Edition) recorded EFS as “sweet, astringent, and flat, attributes to the spleen and kidney meridians, benefiting the kidney and consolidating sperm, tonifying the spleen and inhibiting diarrhoea, eliminating dampness and arresting leucorrhea. It is used for spermatorrhea, enuresis and frequent urination, splenoasthenic diarrhea, and leucorrhea” [9]. In India, EFS is known as makhana and is applied in Ayurvedic medicines for treating diseases including bile disorders, persistent diarrhea, kidney disorders, rheumatic disorders, excessive leucorrhea, hepatic dysfunctioning, etc. [27]. While in Japan, EFS is recorded in Kampo medicine for improving metabolic arthritis, urinary incontinence, and leucorrhea. In addition to EFS, the whole plant of *E. ferox* can be used as food or medicine. As early as the report in the Compendium of Materia Medica in the Ming dynasty, China, the stems, leaves, and roots of *E. ferox* were applied to treat different diseases. To ascertain the efficacy and traditional uses of *E. ferox*, we summarized the records in ancient herbal works or research reports (Table 1).

## 4. Phytochemistry

**Figure 2:**
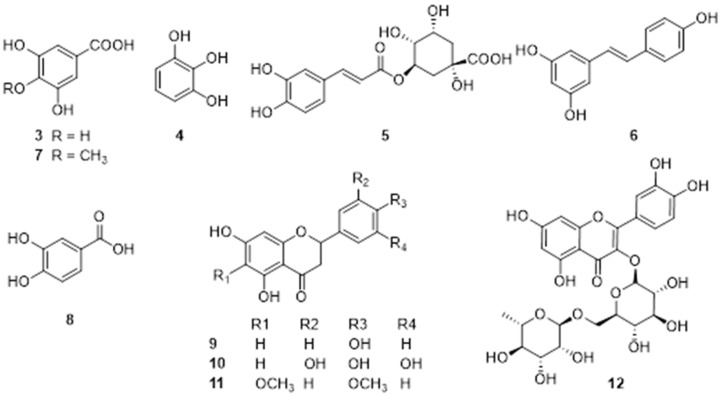
Chemical structures of polyphenols and flavonoids of *E. ferox*.

**Figure 3:**
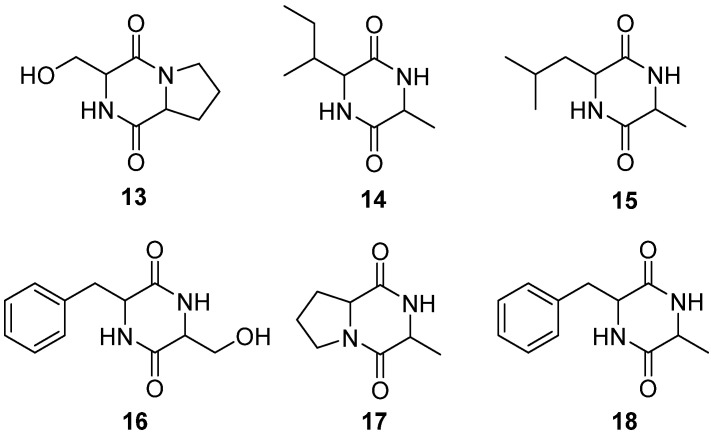
Chemical structures of cyclic peptides of *E. ferox*.

**Figure 4:**
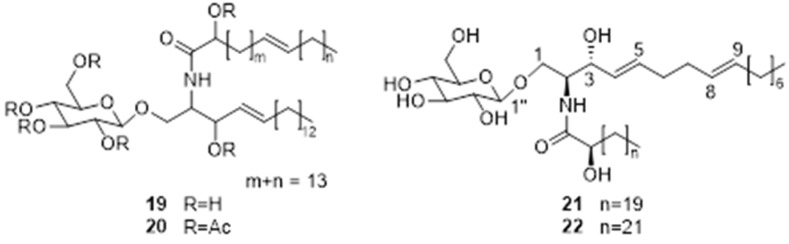
Chemical structures of cerebrosides of *E. ferox*.

**Figure 5:**
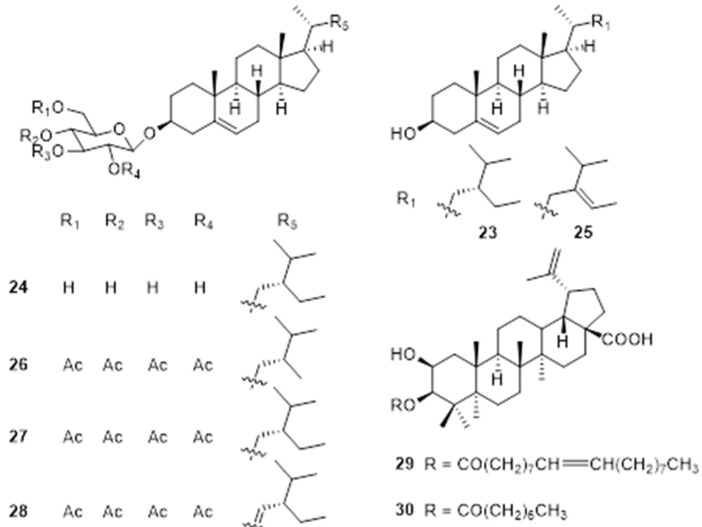
Chemical structures of steroids and pentacyclic triterpenes of *E. ferox*.

**Figure 6:**
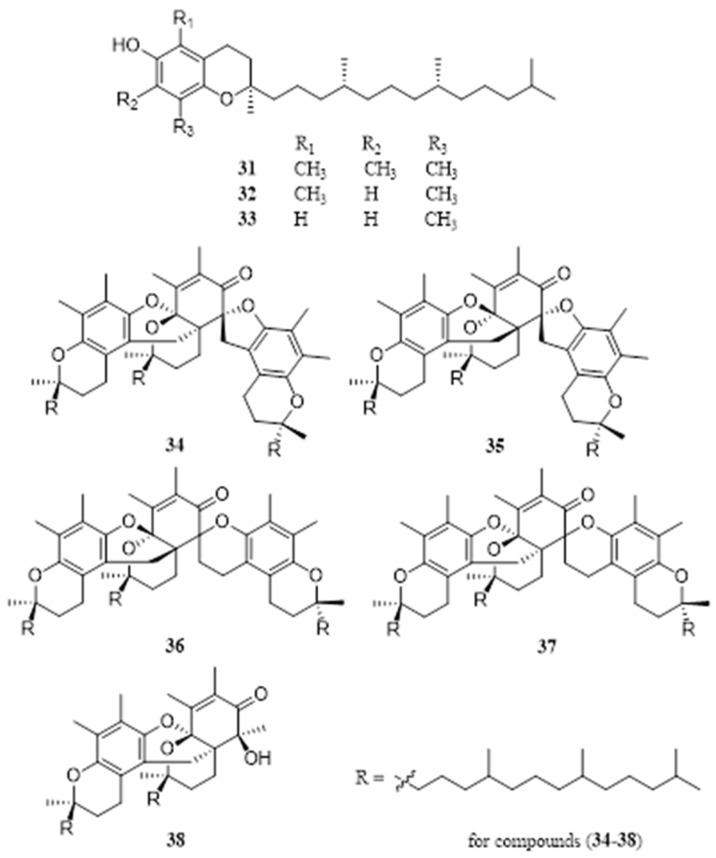
Chemical structures of tocopherols of *E. ferox*.

**Figure 7:**
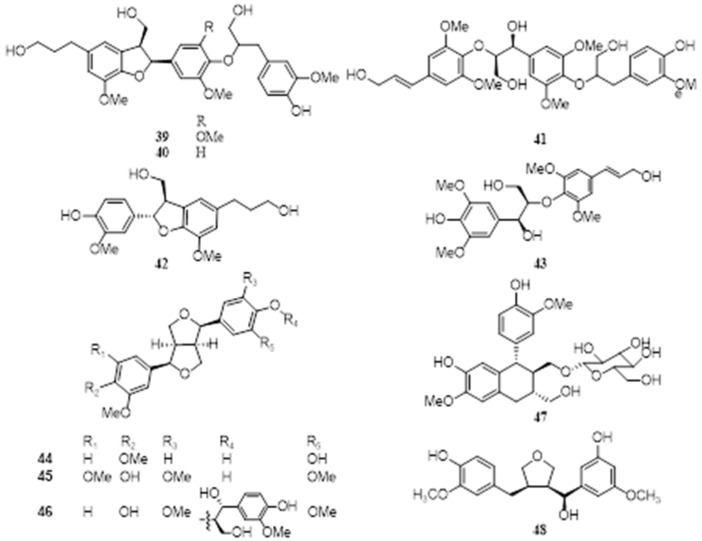
Chemical structures of lignans of *E. ferox*.

**Figure 8:**
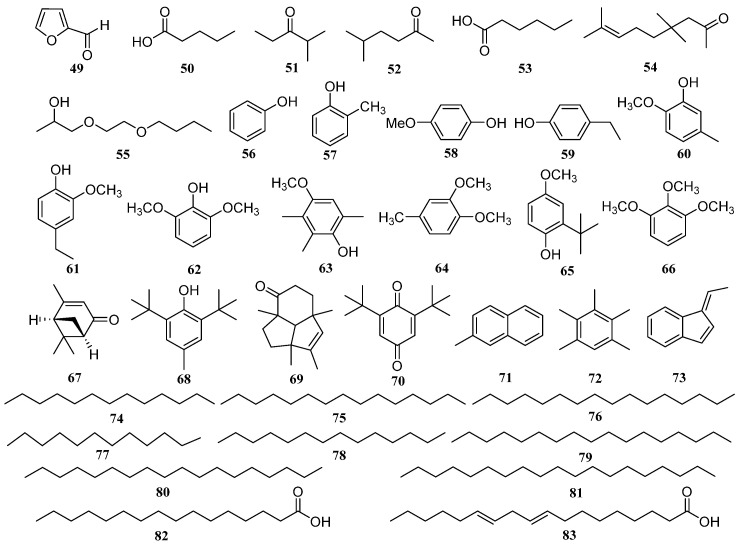
Chemical structures of essential oils of *E. ferox*.

**Figure 9:**
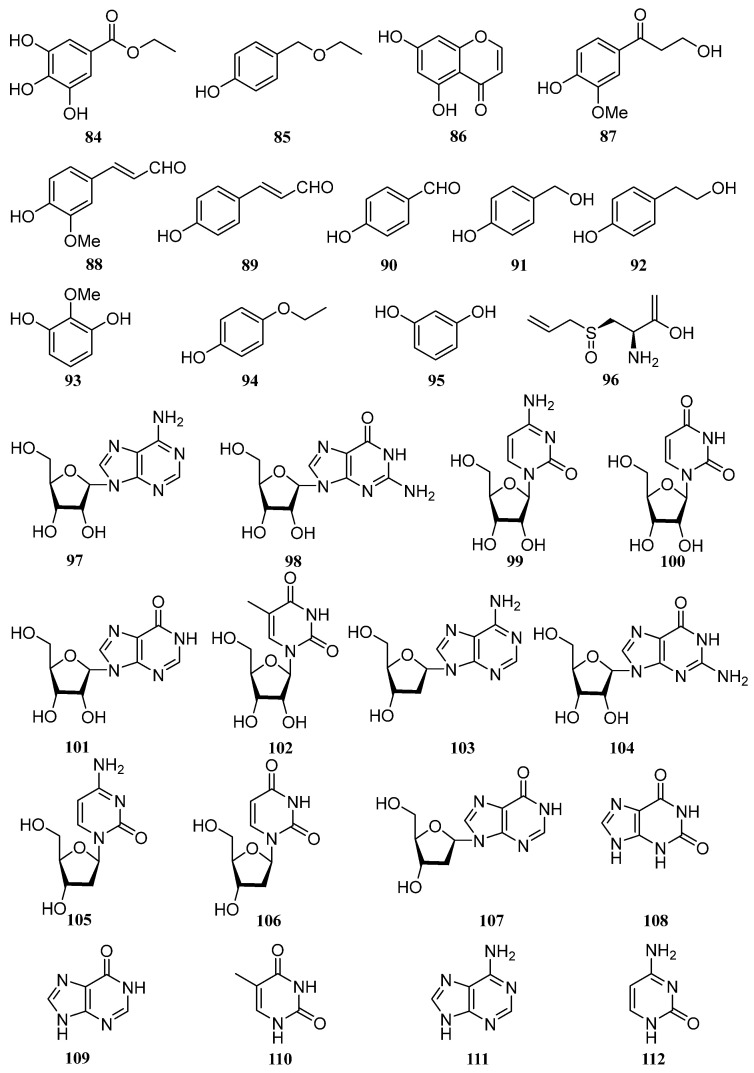
Chemical structures of other compounds of *E. ferox*.

Various phytochemicals have been isolated and determined in *E. ferox,* which can be classified into polysaccharides, polyphenols, flavonoids, cyclic dipeptides, cerebrosides, phytosterol, tocopherols, and triterpenoids based on the chemical properties. Over 100 secondary constituents were tentatively identified from leaves, petioles, fruit peels, seed shells, and kernels of *E. ferox* by UHPLC-MS/MS analysis, with polyphenols occupying 87%. Glycosylated flavonoids are significantly accumulated in the leaves, polyphenols are abundant in the seed, shell, and phenolic acids are predominant in the fruit peel. However, flavonoids vary among the five tissues [30]. All of those reported phytoconstituents are listed in Table 2 in text and their structures are presented in Figure 2, Figure 3, Figure 4, Figure 5, Figure 6, Figure 7, Figure 8 and Figure 9.

### 4.1. Polysaccharides

Polysaccharides are one of the main components of *E. ferox* that exert multiple pharmacological activities [10]. A polysaccharide named EPJ (**1**) was isolated from EFS by DEAE-52 cellulose chromatography and Sephadex G-100 column, which is mainly composed of glucose and rhamnose with a molar ratio of 5.46:1, and the molecular weight was determined to 15.367 kDa [31]. Zhang et al. isolated a novel polysaccharide EFSP-1 (**2**) from EFS by DEAE sepharose FF and Superdex™ 75 gel chromatography, which was mainly composed of (1→4)-α-D-Glcp with branches substituted at O-6 and terminated with T-α-D-Glcp. The structure of EFSP-1 was characterized by NMR, FT-IR, and GC-MS [32]. The high starch content (72.27–83%) made *E. ferox* into a superfood, while resistant starch has gained widespread focus for its physiological functions. A type 3 resistant starch (RS3) was isolated from EFS, and it belongs to B + V type crystal and exerts high thermal stability [33]. Moreover, amylopullulanase-treated *E. ferox* flour promoted the content of resistant starch and inhibited the release of glucose during in vitro digestibility analysis [34].

### 4.2. Polyphenols and Flavonoids

Polyphenols are a class of secondary metabolites with a polyphenolic structure widely present in *E. ferox*, mainly existing in the seed coat, roots, leaves, and fruits. An ultrasonic-assisted extraction technology was performed for the extraction of phenolic compounds from *E. ferox* seed shells, and three polyphenols and one flavonoid were determined by HPLC analysis (**3**–**5**, **12**) (Table 2). In addition, resveratrol (**6**), compound 4-O-methyl gallic acid (**7**), and protocatechuic acid (**8**) were identified from the ethyl acetate extract of EFS. Dihydroflavonoids are the most reported flavonoids in the seeds of *E. ferox* (**9**–**11**). The targeted flavonoid metabolome was determined to explore the dynamic changes of flavonoid biosynthesis by LC-ESI-MS/MS analysis, a total of 129 flavonoid metabolites were identified, including 11 flavanones, 8 dihydroflavanols, 16 flavanols, 29 flavonoids, 3 isoflavones, 12 anthocyanins, 29 flavonols, 6 flavonoid carbosides, 3 chalcones, and 13 proanthocyanidins [35]. However, these compounds were inferred by mass spectrometry and their reliability needs to be further verified. The structures of isolated polyphenols and flavonoids are shown in Figure 2.

**Table 2:** Phytochemical compounds identified from *E. ferox*.

No.	Compounds	Molecular Formula	Type	Plant Part	Analytical Methods	**Ref.**
**1**	EPJ	-	Polysaccharide	Seeds	CC, FT-IR	[31]
**2**	EFSP-1	-	Polysaccharide	Seeds	GPC, FT-IR, GC-MS, NMR	[32]
**3**	Gallic acid	C_7_H_6_O_5_	Polyphenol	Seed shells, Seeds	TLC, MS, NMR, HPLC	[12,16,36]
**4**	Pyrogallol	C_6_H_6_O_3_	Polyphenol	Seed shells	HPLC	[36]
**5**	Chlorogenic acid	C_16_H_18_O_9_	Polyphenol	Seed shells	HPLC	[36]
**6**	Resveratrol	C_14_H_12_O_3_	Polyphenol	Seeds	HPLC	[16]
**7**	4-*O*-methyl gallic acid	C_8_H_8_O_5_	Polyphenol	Seeds	NMR, MS, HPLC	[37]
**8**	Protocatechuic acid	C_7_H_6_O_4_	Polyphenol	Seeds	HPLC	[12]
**9**	Naringenin	C_15_H_12_O_5_	Flavonoid	Seed shells, Seeds	HPLC, GPC, LC-ESI-MS/MS, NMR, FT-IR, UV, IR, HR-EIMS	[18,38,39]
**10**	Dihydrotricetin	C_15_H_12_O_7_	Flavonoid	Seed shells	HPLC, LC-ESI-MS/MS	[18]
**11**	Pectolinarigenin	C_17_H_16_O_6_	Flavonoid	Seeds	HPLC	[12]
**12**	Rutin	C_27_H_30_O_16_	Flavonoid	Seed shells	HPLC	[36]
**13**	Cyclo(Pro-Ser)	C_8_H_12_O_3_N_2_	Cyclodipeptide	Seeds	GPC, NMR, FT-IR, MS	[38]
**14**	Cyclo(Ile-Ala)	C_9_H_16_O_2_N_2_	Cyclodipeptide	Seeds	GPC, NMR, FT-IR, MS	[38]
**15**	Cyclo(Leu-Ala)	C_9_H_16_O_2_N_2_	Cyclodipeptide	Seeds	GPC, NMR, FT-IR, MS	[38]
**16**	Cyclo(Phe-Ser)	C_12_H_14_O_3_N_2_	Cyclodipeptide	Seeds	GPC, NMR, TR, MS	[40]
**17**	Cyclo(Ala-Pro)	C_8_H_12_O_2_N_2_	Cyclodipeptide	Seeds	GPC, NMR, TR, MS	[40]
**18**	Cyclo(Phe-Ala)	C_12_H_14_O_2_N_2_	Cyclodipeptide	Seeds	GPC, NMR, TR, MS	[40]
**19**	N-*α*-hydroxy-*cis*-octadecaenoyl-l-*O*-*β*-glucopyranosylsphingosine	C_42_H_79_O_9_N	Cerebroside	Rhizomes with adventitious root	CC, EI-MS, NMR	[41]
**20**	Peracetylated cerebroside	C_54_H_91_O_15_N	Cerebroside	Rhizomes with adventitious root	CC, EI-MS, NMR	[41]
**21**	(2*S*,3*R*,4*E*,8*E*,2′*R*)-1-*O*-(*β*-glucopyranosyl)-N-(2′-hydroxydocosanoyl)-4,8-sphingadienine	C_44_H_83_O_9_N	Cerebroside	Seeds	CC, MPLC, IR, UV, HRFAB-MS, EI-MS, NMR	[42]
**22**	(2S,3R,4E,8E,2′R)-1-*O*-(*β*-glucopyranosyl)-N-(2′-hydroxytetracosanoyl)-4,8-sphingadienine	C_46_H_87_O_9_N	Cerebroside	Seeds	CC, MPLC, IR, UV, HRFAB-MS, EI-MS, NMR	[42]
**23**	*β*-sitosterol	C_29_H_50_O	Steroid	Seeds	HPLC	[12]
**24**	Daucosterol	C_35_H_60_O_6_	Steroid	Seeds	HPLC	[12]
**25**	Fucosterol	C_29_H_48_O	Steroid	Seeds	HPLC, NMR, MS	[37]
**26**	24-methylcholest-5-enyl-3*β*-*O*-pyranoglucoside	C_43_H_49_O_10_	Steroid	Rhizomes with adventitious root	TLC, CC, NMR, MS	[43]
**27**	24-ethylcholest-5-enyl-3*β*-*O*-pyranoglucoside	C_44_H_51_O_10_	Steroid	Rhizomes with adventitious root	TLC, CC, NMR, MS	[43]
**28**	24-ethylcholesta-5,22*E*-dienyl-3*β*-*O*-pyranoglucoside	C_44_H_49_O_10_	Steroid	Rhizomes with adventitious root	TLC, CC, NMR, MS	[43]
**29**	2*β*-hydroxybetulinic acid 3*β*-caprylate	C_38_H_62_O_5_	Pentacyclic triterpene	Seeds	HPLC, NMR, MS, FT-IR, UV	[44]
**30**	2*β*-hydroxybetulinic acid 3*β*-oleiate	C_48_H_80_O_5_	Pentacyclic triterpene	Seeds	CC, FT-IR, ESI-MS, NMR, UV	[45]
**31**	*α*-tocopherol	C_29_H_50_O_2_	Tocopherol	Seeds	GPC, TLC, NMR, FT-IR, MS, GC-MS, HPLC, UV	[38,46,47]
**32**	*β*-tocopherol	C_28_H_48_O_2_	Tocopherol	Seeds	GPC, TLC, NMR, FT-IR, MS	[38]
**33**	*δ*-tocopherol	C_27_H_46_O_2_	Tocopherol	Seeds	GPC, TLC, NMR, FT-IR, MS	[38]
**34**	Ferotocotrimer C/E	C_86_H_142_O_6_	Tocopherol	Seeds	CC, TLC, MPLC, IR, UV, HRFAB-MS, NMR, HR-ESI-MS, CD	[42,48]
**35**	Ferotocotrimer D	C_86_H_142_O_6_	Tocopherol	Seeds	CC, TLC, MPLC, IR, UV, HRFAB-MS, NMR, HR-ESI-MS, CD	[42]
**36**	Tocopherol trimer IVa	C_87_H_144_O_6_	Tocopherol	Seeds	CC, TLC, MPLC, IR, UV, HRFAB-MS, NMR, HR-ESI-MS, CD	[42]
**37**	Tocopherol trimer IVb	C_87_H_144_O_6_	Tocopherol	Seeds	CC, TLC, MPLC, IR, UV, HRFAB-MS, NMR, HR-ESI-MS, CD	[42]
**38**	Ferotocodimer A	C_58_H_98_O_5_	Tocopherol	Seeds	CC, TLC, MPLC, IR, UV, HRFAB-MS, NMR, HR-ESI-MS, CD	[48]
**39**	Euryalin A	C_31_H_38_O_10_	Lignan	Seeds	CC, HPLC, NMR, UV, IR, HR-EIMS, ESIMS	[39]
**40**	Euryalin B	C_30_H_36_O_9_	Lignan	Seeds	CC, HPLC, NMR, UV, IR, HR-EIMS, ESIMS	[39]
**41**	Euryalin C	C_32_H_40_O_12_	Lignan	Seeds	CC, HPLC, NMR, UV, IR, HR-EIMS, ESIMS	[39]
**42**	rel-(2*R*,3*β*)-7-*O*-methylcedrusin	C_20_H_24_O_6_	Lignan	Seeds	CC, HPLC, NMR, UV, IR, HR-EIMS, ESIMS	[39]
**43**	syringylglycerol-8-*O*-4-(sinapyl alcohol) ether	C_23_H_30_O_9_	Lignan	Seeds	CC, HPLC, NMR, UV, IR, HR-EIMS, ESIMS	[39]
**44**	(1*R*,2*R*,5*R*,6*S*)2-(3,4-dimethoxyphenyl)-6-(3,4-dihydroxyphenyl)-3,7-dioxabicyclo [3.3.0]octane	C_20_H_22_O_6_	Lignan	Seeds	CC, HPLC, NMR, UV, IR, HR-EIMS, ESIMS	[39]
**45**	(+)-syringaresinol	C_22_H_26_O_8_	Lignan	Seeds	CC, HPLC, NMR, UV, IR, HR-EIMS, ESIMS	[39]
**46**	Buddlenol E	C_31_H_36_O_11_	Lignan	Seeds	CC, HPLC, NMR, UV, IR, HR-EIMS, ESIMS	[18,39]
**47**	(+)-Isolariciresinol 9’-*O*-glucoside	C_26_H_34_O_11_	Lignan	Seeds	GPC, TLC, NMR, FT-IR, MS	[38]
**48**	3-(4-hydroxy-3-methoxybenzyl)-4-[(7’*R*),5’-dihydroxy-3’-methoxybenzyl] tetrahydrofuran	C_20_H_24_O_6_	Lignan	Seeds	HPLC, NMR, MS	[37]
**49**	Furfural	C_5_H_4_O_2_	Volatile constituents	Seeds	GC-MS	[46]
**50**	Pentanoic acid	C_5_H_10_O_2_	Volatile constituents	Seeds	GC-MS	[46]
**51**	2-methyl-3-pentanone	C_6_H_12_O	Volatile constituents	Seeds	GC-MS	[46]
**52**	5-methyl-2-furancarboxaldehyde	C_7_H_14_O	Volatile constituents	Seeds	GC-MS	[46]
**53**	Hexanoic acid	C_6_H_12_O_2_	Volatile constituents	Seeds	GC-MS	[46]
**54**	4, 4, 8-trimethyl-non-7-en-2-one	C_12_H_22_O	Volatile constituents	Seeds	GC-MS	[46]
**55**	1-(2-butoxyethoxy)-2-propanol	C_9_H_20_O_3_	Volatile constituents	Seeds	GC-MS	[46]
**56**	Phenol	C_6_H_6_O	Volatile constituents	Seeds	GC-MS	[46]
**57**	2-methylphenol	C_7_H_8_O	Volatile constituents	Seeds	GC-MS	[46]
**58**	4-methylphenol	C_7_H_8_O_2_	Volatile constituents	Seeds	GC-MS	[46]
**59**	4-ethylphenol	C_8_H_10_O	Volatile constituents	Seeds	GC-MS	[46]
**60**	Isocreosol		Volatile constituents	Seeds	GC-MS	[46]
**61**	4-ethylguaiacol	C_9_H_12_O_2_	Volatile constituents	Seeds	GC-MS	[46]
**62**	2, 6-dimethoxyphenol	C_8_H_10_O_3_	Volatile constituents	Seeds	GC-MS	[46]
**63**	4-methoxy-2, 3, 6-trimethylphenol	C_10_H_14_O_2_	Volatile constituents	Seeds	GC-MS	[46]
**64**	3, 4-dimethoxytoluene	C_9_H_12_O_2_	Volatile constituents	Seeds	GC-MS	[46]
**65**	3-tert-butyl-4-hydroxyanisole	C_11_H_16_O_2_	Volatile constituents	Seeds	GC-MS	[46]
**66**	1, 2, 3-trimethoxybenzene	C_9_H_12_O_3_	Volatile constituents	Seeds	GC-MS	[46]
**67**	[3.1.1] hept-3-en-2-one, 4, 6, 6-trimethyl-bicyclo	C_10_H_14_O	Volatile constituents	Seeds	GC-MS	[46]
**68**	Butylated hydroxytoluene	C_15_H_24_O	Volatile constituents	Seeds	GC-MS	[46]
**69**	1*S*, 4*R*, 7*R*, 11*R*-1, 3, 4, 7-tetramethyltricyclo [5.3.1.0(4, 11)] undec-2-en-8-one	C_15_H_22_O	Volatile constituents	Seeds	GC-MS	[46]
**70**	2, 6-bis (1, 1-dimethylethyl)-2, 5-cyclohexadiene-1, 4-dione	C_14_H_20_O_2_	Volatile constituents	Seeds	GC-MS	[46]
**71**	2-methylnaphthalene	C₁₁H₁₀	Volatile constituents	Seeds	GC-MS	[46]
**72**	Pentamethyl benzene	C_11_H_16_	Volatile constituents	Seeds	GC-MS	[46]
**73**	1-ethylidene-1H-indene	C_11_H_10_	Volatile constituents	Seeds	GC-MS	[46]
**74**	Tridecane	C_13_H_28_	Volatile constituents	Seeds	GC-MS	[46]
**75**	Pentadecane	C_15_H_32_	Volatile constituents	Seeds	GC-MS	[46]
**76**	Hexadecane	C_16_H_34_	Volatile constituents	Seeds	GC-MS	[46]
**77**	Dodecane	C_12_H_26_	Volatile constituents	Seeds	GC-MS	[46]
**78**	Tetradecane	C_14_H_30_	Volatile constituents	Seeds	GC-MS	[46]
**79**	Heptadecane	C_17_H_36_	Volatile constituents	Seeds	GC-MS	[46]
**80**	Octadecane	C_18_H_38_	Volatile constituents	Seeds	GC-MS	[46]
**81**	Nonadecane	C_19_H_40_	Volatile constituents	Seeds	GC-MS	[46]
**82**	Palmitic acid	C_16_H_32_O_2_	Volatile constituents	Seeds	GC-MS	[46]
**83**	Linoleic acid	C_18_H_32_O_2_	Volatile constituents	Seeds	GC-MS	[46]
**84**	Ethyl gallate	C_9_ H_10_O_5_	Ester	Seeds	HPLC	[12]
**85**	4-hydroxybenzylethyl ether	C_8_H_10_O_2_	Ether	Seeds	CC, HPLC, NMR, UV, IR, HR-EIMS, ESIMS	[39]
**86**	5,7-dihydroxychromone	C_9_H_6_O_4_	Ketone	Seeds	HPLC	[12]
**87**	*ω*-hydroxypropioguaiacone	C_10_H_12_O_4_	Ketone	Seeds	CC, HPLC, NMR, UV, IR, HR-EIMS, ESIMS	[39]
**88**	Coniferyl aldehyde	C_10_H_10_O_3_	Aldehyde	Seeds	CC, HPLC, NMR, UV, IR, HR-EIMS, ESIMS	[39]
**89**	Trans-*p*-hydroxycinnamaldehyde	C_9_H_8_O_2_	Aldehyde	Seeds	CC, HPLC, NMR, UV, IR, HR-EIMS, ESIMS	[39]
**90**	*p*-hydroxybenzaldehyde	C_7_H_6_O_2_	Aldehyde	Seeds	CC, HPLC, NMR, UV, IR, HR-EIMS, ESIMS	[39]
**91**	*p*-hydroxybenzyl alcohol	C_7_H_8_O_2_	Alcohol	Seeds	CC, HPLC, NMR, UV, IR, HR-EIMS, ESIMS	[39]
**92**	*p*-hydroxyphenethyl alcohol	C_8_H_10_O_2_	Alcohol	Seeds	CC, HPLC, NMR, UV, IR, HR-EIMS, ESIMS	[39]
**93**	2-methoxybenzene-1,3-diol	C_7_H_8_O_3_	Phenyl alcohol	Seeds	CC, HPLC, NMR, UV, IR, HR-EIMS, ESIMS	[39]
**94**	4-ethoxyphenol	C_8_H_10_O_2_	Phenol	Seeds	CC, HPLC, NMR, UV, IR, HR-EIMS, ESIMS	[39]
**95**	Resorcinol	C_6_H_6_O_2_	Phenol	Seeds	HPLC, NMR, MS	[37]
**96**	Alliin	C_6_H_11_NO_3_S	Sulfoxide	Seeds	HPLC	[16]
**97**	Adenosine	C_10_H_13_N_5_O_4_	Nucleoside	Seeds	HPLC-ESI-TQ-MS/MS	[49]
**98**	Guanosine	C_10_H_13_N_5_O_5_	Nucleoside	Seeds	HPLC-ESI-TQ-MS/MS	[49]
**99**	Cytidine	C_9_H_13_N_3_O_5_	Nucleoside	Seeds	HPLC-ESI-TQ-MS/MS	[49]
**100**	Uridine	C_9_H_12_N_2_O_6_	Nucleoside	Seeds	HPLC-ESI-TQ-MS/MS	[49]
**101**	Inosine	C_10_H_12_N_4_O_5_	Nucleoside	Seeds	HPLC-ESI-TQ-MS/MS	[49]
**102**	Thymidine	C_10_H_14_N_2_O_5_	Nucleoside	Seeds	HPLC-ESI-TQ-MS/MS	[49]
**103**	2′-deoxyadenosine	C_10_H_13_N_5_O_3_	Nucleoside	Seeds	HPLC-ESI-TQ-MS/MS	[49]
**104**	2′-deoxyguanosine	C_10_H_13_N_5_O_4_	Nucleoside	Seeds	HPLC-ESI-TQ-MS/MS	[49]
**105**	2′-deoxycytidine	C_9_H_13_N_3_O_4_	Nucleoside	Seeds	HPLC-ESI-TQ-MS/MS	[49]
**106**	2′-deoxyuridine	C_9_H_12_N_2_O_5_	Nucleoside	Seeds	HPLC-ESI-TQ-MS/MS	[49]
**107**	2′-deoxyinosine	C_10_H_12_N_4_O_4_	Nucleoside	Seeds	HPLC-ESI-TQ-MS/MS	[49]
**108**	Xanthine	C_5_H_4_N_4_O_2_	Nucleobase	Seeds	HPLC-ESI-TQ-MS/MS	[49]
**109**	Hypoxanthine	C_5_H_4_N_4_O	Nucleobase	Seeds	HPLC-ESI-TQ-MS/MS	[49]
**110**	Thymine	C_5_H_6_N_2_O_2_	Nucleobase	Seeds	HPLC-ESI-TQ-MS/MS	[49]
**111**	Adenine	C_5_H_5_N_5_	Nucleobase	Seeds	HPLC-ESI-TQ-MS/MS	[49]
**112**	Cytosine	C_4_H_5_N_3_O	Nucleobase	Seeds	HPLC-ESI-TQ-MS/MS	[49]

### 4.3. Cyclic Peptides

Cyclic peptides are a kind of cyclo-compounds composed of common and uncommon amino acids. Due to specific properties, such as good target selectivity, binding affinity, and low toxicity, cyclic peptides have become attractive lead compounds for drug development [50]. Multiple bioactive activities have been reported including antimicrobial, anti-infection, anti-tumors, anti-chronic kidney diseases, anti-diabetes, and memory improvement [51,52]. Using thin-layer in situ chemical reactions, several cyclic dipeptides (**13**–**18**) have been isolated from EFS. The isolated cyclic peptides are shown in Table 2 and Figure 3.

### 4.4. Cerebrosides

Cerebrosides are neutral chemicals that consist of a monosaccharide and ceramide bound by a *β*-glycosidic bond to the C1 of esfingol. As an important component of cell membranes in the nervous system, cerebrosides play critical roles in regulating membrane dynamics and forming internal structures. Four novel cerebrosides have been elucidated in the rhizome with the adventitious root of *E. ferox* (**19**, **20**) and EFS (**21**, **22**), respectively [41,42]. The structures of cerebrosides are shown in Figure 4.

### 4.5. Steroids and Pentacyclic Triterpenes

Steryl glycosides (SGs) and acylated steryl glycosides (ASGs) are two major derivatives of sterols. Steroidal glycolipids are considered to be unique glycolipids that play an important role in the structure of cells. Three sterols, *β*-sitosterol (**23**), daucosterol (**24**), and fucosterol (**25**) were isolated from EFS ethyl acetate extract [12,37]. While, three glucosylsterols named 24-methylcholest-5-enyl-3*β*-*O*-pyranoglucoside (**26**), 24-ethylcholest-5-enyl-3*β*-*O*-pyranoglucoside (**27**), and 24-ethylcholesta-5,22*E*-dienyl-3*β*-*O*-pyranoglucoside (**28**) were obtained from the rhizomes with adventitious roots of *E. ferox* [43].

Triterpenoids are of great interest to researchers owing to their wide range of biological activities. Gong determined the contents of triterpenoids in 70% ethanol extract of *E. ferox* seed shell using vanillin-perchloric acid method, the total triterpenoids are up to 36.7% [53]. In order to investigate the putative active compounds responsible for antidiabetic, antioxidant, and antihyperlipidemic in EFS, Ahmed et al. obtained two novel triterpenoids, 2*β*-hydroxybetulinic acid 3*β*-oleiate (**29**) and 2*β*-hydroxybetulinic acid 3*β*-caprylate (**30**), from the ethyl acetate extract [44,45]. The specific information and structures of each compound are shown in Table 2, Figure 5.

### 4.6. Tocopherol

Tocopherols are a series of organic chemicals consisting of various methylated phenols. EFS contains an extraordinarily high content of tocopherols, which may contribute to scavenging free radicals and antioxidant effects. After extraction with 95% methanol and purified with silica gel and Sephadex LH-20, Li et al. identified three tocopherols, α-tocopherol (**31**), *β*-tocopherol (**32**), and *δ*-tocopherol (**33**), from EFS [38]. In a search for bioactive constituents of EFS, Rowet al. identified two new tocopherol trimers, ferotocotrimer C (**34**) and D (**35**), and two known tocopherol trimers, IVb (**36**) and IVa (**37**) from EFS (16.1 kg) methanol refluxed extracts, their structures were determined on the basis of spectroscopic data, especially 1D and 2D NMR experiments [42]. In addition, two new tocopherol polymers, the chroman-type dimer ferotocodimer A (**38**) and the spiro-type trimer ferotocotrimer E (**34**), were isolated from EFS [48] (Figure 6). Their structures were determined based on spectroscopic data, especially 1D and 2D NMR analysis. The absolute configuration of **34** and **38** were determined by CD exciton chirality and ROESY experiment.

### 4.7. Lignans

Song et al. extracted EFS (50 kg) with 85% EtOH three times, the extract (1.2 kg) was suspended in water, and was successively partition with EtOAc, the components in the EtOAc extract of EFS (55 g) were investigated, 3 new sesquineolignans, named euryalins A-C (**39**–**41**), 2 neolignans, named rel-(2α,3*β*)-7-*O*-methylcedrusin (**42**) and syringylglycerol-8-*O*-4-(sinapyl alcohol) ether (**43**), and 3 furofuran-type lignans, named (1*R*,2*R*,5*R*,6*S*)2-(3,4-dimethoxyphenyl)-6-(3,4-dihydroxyphenyl)-3,7-dioxabicyclo [3.3.0]octane (**44**), (+)-syringaresinol (**45**), and buddlenol E (**46**), were identified. The relative configuration was determined by the ROESY while the absolute configuration remained undetermined [39]. In addition, another 2 antioxidative lignans, (+)-Isolariciresinol 9′-*O*-glucoside (**47**) and 3-(4-hydroxy-3-methoxybenzyl)-4-[(7′*R*),5′-dihydroxy-3′-methoxybenzyl]tetrahydrofuran (**48**), were also isolated from EFS [37,38]. The isolated lignans are shown in Table 2, and the corresponding structures are shown in Figure 7.

### 4.8. Volatile Constituents

A Clevenger’s apparatus was used for the isolation of essential oil by hydro-distillation for 6 h in EFS with a yield of 0.028% (*v*/*w*). A total of 37 components were identified by gas chromatography-mass spectroscopy. Compounds were identified by comparison of their retention indices and mass spectra with the data stored in the National Institute of Standards and Technology (NIST 05). The main constituents were butylated hydroxytoluene (**49**) (38.7%), palmitic acid (**50**) (11.0%), linoleic acid (**51**) (9.0%), and hexanoic acid (**53**) (3.9%) [46]. The structures of volatile constituents (**49**–**83**) are shown in Figure 8.

### 4.9. Others

Other phytochemicals reported in *E. ferox* include esters (**84**, **85**), ketones (**86**, **87**), aldehydes (**88**–**90**), alcohols (**91**, **92**), phenols (**93**, **94**, **95**), and sulfoxide (**96**). In addition, 16 nucleosides and nucleobases (**97**–**112**) were simultaneously determined by HPLC-ESI-TQ-MS/MS, and their contents of them in 26 batch samples were quantified with standards [49] (Figure 9). An ultrasound-assisted technique was established for the extraction of anthocyanins from the waste leaves of *E. ferox*, and the yield of anthocyanins was 2.82 ± 0.03 mg/g. Nineteen anthocyanins were identified by HPLC-QTOF-MS/MS [26].

## 5. Pharmacological Activities

**Figure 10:**
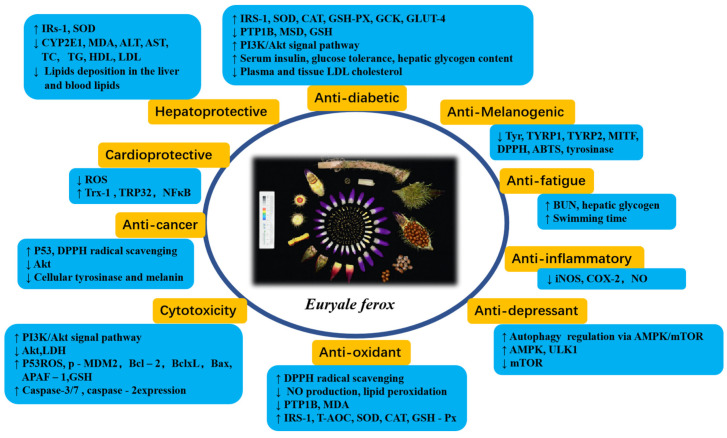
The summarized pharmacological mechanism and effects of *E. ferox.* Up arrow symbol means upregulation and down arrow symbol means downregulation.

Various in vitro and in vivo studies have indicated that *E. ferox*-derived extracts and phytoconstituents exhibited antioxidant and antidiabetic effects. In addition, the anti-tumor, anti-hyperlipidaemic, antibacterial, anti-inflammatory, antimelanogenic, antiaging, antifatigue, cardioprotective, and hepatoprotective activities have also been reported. The pharmacological properties of *E. ferox* were summarized in Table 3 and Figure 10.

### 5.1. Antioxidant and Anti-Inflammatory Activity

The evaluation of antioxidant activity can be performed in three main ways including directly 1,1-diphenyl-2-picrylhydrazyl (DPPH) and reactive oxygen species (ROS) scavenge, the activation of antioxidant enzymes such as catalase (CAT), superoxide dismutase (SOD) and glutathione peroxidase (GSH-Px), and improvement of somatic cellular integrity. The methanol, ethanol, and aqueous extracts of EFS showed DPPH scavenging effects, while the methanol extract showed anti-inflammatory activity in RAW 264.7 cell lines [19,20,21,39]. A corilagin monomer was isolated and identified from *E. ferox* shell, and showed an anti-inflammatory effect against LPS-induced Raw264.7 cells, the level of NO, TNF-α, IL-6, and IL-1*β* were significantly reduced, while the mechanism was related to NF-κB and MAPK signaling pathway [17].

EFS methanol extracts exerted high levels of DPPH radical scavenging activity, lipid peroxidation inhibition, protection of H_2_O_2_-induced apoptosis, and antioxidant enzyme activity enhancement. Among various fractionated samples of *E. ferox*, the ethyl acetate and butanol fractions exhibited relatively high antioxidative activity [20]. The essential oil from the EFS exhibited strong DPPH and ABTS scavenging activity, the IC_50_ of which were 6.27 ± 0.31 and 2.19 ± 0.61 μg/mL, respectively [46]. Fermentation of *E. ferox* with *Lactobacillus curvatus* increases the content of the various bioactive components including smaller molecular weights of polysaccharides and polypeptides, enhances its antioxidant capacity, and attenuates oxidative stress-induced human skin fibroblast apoptosis and senescence [21]. In addition, the phenolic extracts from the *E. ferox* seed shells and anthocyanins from the *E. ferox* leaves showed DPPH and 2,2′-azino-bis(3-ethylbenzothiazoline-6-sulfonic acid) (ABTS) scavenging effects [18,26,36].

Cell wall polysaccharides (EFPP) were isolated from the petioles and pedicels of *E. ferox* using the DEAE-52 column, and four major fractions (EFPP-1, EFPP-2, EFPP-3, and EFPP-4) were obtained. The crude EFPP and EFPP-4 could effective against H_2_O_2_-induced injury on HUVEC and VSMC through enhancement of T-AOC, SOD, and CAT activities and decrease of MDA content [55].

The anti-oxidative activities vary among different parts of *E. ferox*, while the seed extracts showed better effects than seed shells, leaves, petioles, and pedicels from the aforementioned reports. Further studies should aim to purify and characterize the active phytoconstituents from the antioxidative extracts.

### 5.2. Antidiabetic and Hypoglycemic Activity

The *E. ferox* ethanol extract protected β-cells against ROS-mediated damage by increasing the expression of antioxidant enzymes and reducing hyperglycemia, possibly due to the release of insulin from residual and recovered β-cells in the pancreas of streptozotocin-induced diabetic rats [15]. Another study indicated that germinated EFS extract contained more gentisic acid, caffeic acid, and other 27 effective polyphenols than EFS, corresponding to the higher improved antioxidant and renal indexes, and a more stable effect in regulating the AMPK/mTOR and Keap1/Nrf2/HO-1 signaling pathways, leading to the more attenuated antidiabetic effects [56]. A polysaccharide obtained from EFS, EFSP-1, could increase glucose consumption by up-regulating the expression of GLUT-4 via activating PI3K/Akt signal pathway in insulin resistance HepG2 and 3T3-L1 cells [32]. The antidiabetic activities of two triterpenoids in *E. ferox* were investigated in streptozotocin-induced Wistar rats over a four-week period. After 45 days of gavage consisting of 2*β*-hydroxybetulinic acid 3*β*-caprylate (HBAC) and 2*β*-hydroxybetulinic acid 3*β*-oleiate (HBAO) in diabetic mice, the plasma glucose and insulin were normalized, pancreatic β-cell, the histological architecture of pancreas, kidney, and liver were restored, as well as the endogenous antioxidant enzymes [44,45]. The aforementioned studies suggest that extract of EFS could be an important source of natural antioxidants with hypoglycaemic and hypolipidaemic effects, and could be used as a food additive or functional food in the future.

Another study investigated the extract of Gorgon fruit as a food additive and found that *E. ferox* shell extract (EFSSE) had a significant effect on the in vitro digestibility of bread starch and that EFSSE (2%) fortified bread and exhibited a strong glycemic index inhibition. In addition, the IC_50_ of EFSSE on α-amylase and α-glucosidase inhibitory effect was 62.95 and 52.06 μg/mL, respectively [57]. The hypoglycemic and hypolipidemic effects of triterpenoid-rich 75% ethanol extracts of *E. ferox* shell were investigated in streptozotocin-induced diabetic mice. Gavage of 400 and 600 mg/kg *E. ferox* shell extract for 4 weeks significantly restored the body weight, blood glucose, and insulin resistance [53]. Triterpenoid-rich *E. ferox* shell extract in drinking water (500 mg/L) for 4 weeks significantly attenuated streptozotocin-induced high blood glucose, pancreas injury, higher tyrosine phosphatase-1B level, and low insulin receptor substrate expression [58]. Therefore, *E. ferox* shell extract can be used as a therapeutic ingredient for diabetes induced by insulin resistance.

Crude polysaccharides (EFPP) were prepared from the petioles and pedicels of *E. ferox*, which had a total carbohydrate of 65.72 ± 2.81%, the monosaccharide compositions were Man, GlcA, Rha, Glc, Gal, and Ara at a molar ratio of 0.12:0.01:9.57:0.41:1.00:0.24. After oral administration with EFPP (400 mg/kg) for 28 days, the activities of CAT, SOD and GSH-Px, and MDA contents in the kidney and liver of alloxan-induced mice were significantly ameliorated, as well as the damaged pancreas, kidney, and liver tissues. The blood glucose level was reduced and the serum insulin level was remarkably increased [60].

Currently, network pharmacology as an emerging discipline has been gradually applied to the mechanistic study of phytopharmaceuticals. This method is suitable for multi-component and multi-target studies by using the database of ingredients, targets, and genes to elucidate the complex mechanism of action of a drug in a holistic way. Some investigations have preliminarily elucidated the anti-diabetic mechanism of action of *E. ferox* based on network pharmacology and molecular docking. Twenty-four components of *E. ferox* and 72 targets were identified, of which 9 (FABP1, JUN, LPL, PPARA, TP53, TGFB1, IL1A, MAPK1, CTNNB1) are clinically relevant and mainly regulated by transcription factors such as HNF4A and PPARG. The main components are oleic acid, which targets the proteins encoded by PPARA, LPL, and FABP1, and vitamin E, which binds to the proteins encoded by MAPK1 and TGFB1 [59].

In conclusion, *E. ferox* can be used to treat diabetes mainly through anti-inflammatory, reducing pancreatic β-cell damage and apoptosis, promoting glucose absorption and utilization, and improving insulin resistance and complications. Although noteworthy antidiabetic properties have been attributed to *E. ferox* polysaccharides or triterpenoids, the homopolysaccharide has not been identified, and whether there are any other phytoconstituents responsible for this activity remains to be elucidated. Meanwhile, further clinical validation of the above findings is still needed in conjunction with experiments.

### 5.3. Hepatoprotective and Cardioprotective Activity

Oral administration of the *E. ferox* seed coat ethanol extract (EFSCE) to high-fat diet (HFD)-induced ICR mice at doses of 15 and 30 mg/kg for 4 weeks resulted in a significant reduction in body weight, lipid deposition in the liver and blood lipids. EFSCE also prevented excessive production of MDA and enhanced SOD activity to counteract oxidative stress. In addition, EFSCE was effective in reducing alanine aminotransferase (ALT) and aspartate aminotransferase (AST) activities in HFD-induced mice. EFSCE can be used as a biologically active natural product for the treatment of HFD-induced NAFLD by modulating IRs-1 and CYP2E1 to eliminate lipid accumulation and oxidative stress [13].

Another study investigated if *E. ferox* seeds could reduce myocardial ischemic reperfusion injury. The isolated rat hearts ischemia and reperfusion acute model was constructed to evaluate the cardioprotective effect of *E. ferox* extract (25, 125 or 250 μg/mL), 125 or 250 μg/mL *E. ferox* extract treatment significantly enhanced aortic flow and reduced the infarct size. *E. ferox* (250 and 500 mg/kg/day) oral administration for 21 days improved post-ischemic ventricular function and reduced myocardial infarct size in a chronic ischemic reperfusion model. Two cardioprotective proteins, TRP32, and thioredoxin, were significantly increased. Taken together, this study demonstrated the cardioprotective properties of Makhana and the effects may be related to its upregulation of TRP32 and Trx-1 proteins and ROS scavenge activities [14].

### 5.4. Cytotoxic and Anticancer Activity

The apoptotic effects of EFS ethanol extract (ESE) in A549 lung cancer cells were investigated, ESE induces apoptosis via the regulation of mitochondrial outer membrane potential and generation of ROS. ESE-induced A549 apoptosis is in a p53-dependent manner, in addition, ESE suppressed tumor growth in Balb/c-nu mice bearing A549 xenografts and activated p53 protein [16]. *E. ferox* seed shell extracts (200 μg/mL) showed an inhibitory effect on SGC7901 and HepG2 cell proliferation, with the inhibition rate being 92.63% and 72.40%, respectively. *E. ferox* seed shell extracts (200–800 μg/mL) arrest SGC7901 cells in the G0/G1 phase, and 50–200 μg/mL *E. ferox* seed shell extracts arrest HepG2 cells in the S phase. Meanwhile, the cell mitochondrial membrane potential was significantly reduced and the intracellular calcium influx was increased [61]. Treatment of melan-a cells with 30 μg/mL EFS ethyl acetate fraction produced a strong inhibition of cellular tyrosinase and melanin synthesis, and the lysosomal degradation of tyrosinase was involved in melanogenesis inhibition [23]. Resorcinol (**95**) inhibited melanin synthesis in B16F10 melanoma cells with an IC50 value of 492.8 μM [37].

Two cerebrosides, ferocerebrosides A and B, were isolated from the methanol extract of EFS, and they showed marginal toxicity against brine shrimp with LC50 values of 0.17 and 0.20 mM, respectively [42]. The toxicity study of a new glucan EFSP-1, obtained from EFS, was performed on HepG2 and 3T3-L1 cells, and no obvious toxicity was observed at doses between 100 and 400 μg/mL [32]. Neuroprotective effect of EFS subfractions against glutamate-induced cytotoxicity in hybridoma cells N18-RE-105 was investigated. The EFS ethanolic extract showed a dose-dependent protective effect against 20 mM glutamate-induced neuronal cell death. EFS ethanolic extract was subfractionated with hexane, diethyl ether, and ethyl acetate, the hexane fraction showed the strongest neuroprotective effect against glutamate-induced N18-RE-105 cells. The results suggest that EFS can be used as chemotherapeutic agents in the treatment of neurological disorders [62].

### 5.5. Antifatigue Activity

An exertional swimming test was performed to evaluate the anti-fatigue effect. The phenolic extracts of *E. ferox* can prolong the average duration of exertional swimming, the expression of BUN was significantly reduced, while hepatic glycogen content was dramatically increased. In addition, three main phenolic compounds in the extract were identified as 5,7-dihydroxy-2-(3,4,5-trihydroxyphenyl)-chroman-4-one, naringenin, and buddlenol E [18].

Studies have shown that *E. ferox* is a potential and readily available source of natural antioxidants and has the potential to be a new functional anti-fatigue food or drug. In the future, studies on the chemical composition and safety evaluation of phenolic extract need to be continued with a view to providing valuable information for novel functional food development.

### 5.6. Anti-Depressant Activity

The potential antidepressant effects of EFS petroleum ether fraction (ES-PE) were investigated in a mouse model of chronic unpredictable mild stress (CUMS). Deficits in the open field test, sucrose preference test, tail suspension test, and forced swimming test were observed in mice following CUMS and were reversed following ES-PE administration. ES-PE significantly up-regulated phosphorylation of adenosine monophosphate-activated protein kinase (AMPK) and mammalian autophagy initiating kinase (ULK1) at Ser317, and the ration of p-mTOR/mTOR was suppressed by ES-PE treatment. In addition, ES-PE treatment significantly attenuated Compound C, an inhibitor of AMPK, induced autophagy suppression. GC-MS analysis revealed high levels of vitamin E acetate in ES-PE, suggesting the potential role of VE in the antidepressant effect of ES-PE [22]. Further studies are needed to explore the antidepressant mechanism of ES-PE, in addition to autophagy, as well as other potential phytochemicals.

### 5.7. Other Activities

*E. ferox* seed methanolic extracts exhibit significant antibacterial activity against *Staphylococcus aureus* ATCC 25923, *Escherichia coli* ATCC 25922, and *Pseudomonas aeruginosa* ATCC 27853, the minimum inhibitory concentration (MIC) was 64, 128, and 64 mg/L, respectively [63]. Using the agar cup method, ethyl acetate and ethanol extract of *E. ferox* seed coat showed a higher inhibition zone against *E. coli* and *S. aureus* [64]. In addition, the methanolic *E. ferox* seed and leaf extracts showed anti-fungal effects against *Candida albicans* and *Pencillium notatum* strains [65].

## 6. Toxicity

The non-toxic characteristic of EFS was clearly stated thousands of years ago in Shennong’s Herbal Classic [5]. According to Chinese Pharmacopoeia, the medicinal dosage of EFS is generally 9–15 g per day [13]. If consumed excessively, it may lead to gastrointestinal overload, as EFS contains a lot of starch, protein, and other ingredients that have a solid and astringent effect [34,57]. The “Suixiju Dietary Recipes” records the contraindications of EFS as “EFS is not recommended in the following conditions, including before and after cold affection, malaria, dysentery and hemorrhoids, red urine and constipation, transport failure of the spleen, and postpartum period”. In the modern toxicity evaluations, 24 h incubation of EFS essential oil showed toxicity with an LC50 value of 11.48 ± 0.51 μg/mL in brine shrimp bioassay [46]. Compounds **21** and **22** also showed cytotoxicity in the brine shrimp lethality bioassay, with LC50 values of 0.17 and 0.20 mM, respectively [42]. In another study, oral administration of 70% ethanol EFS extract to Wistar rats for 45 days at doses of 100–400 mg/kg, no lethality and toxicity were found [15]. Nevertheless, the monitoring of adverse reactions to EFS should be further strengthened to improve its safety in clinical application.

## 7. Conclusions and Perspectives

The current study summarized the investigations of *E. ferox* in terms of traditional uses, phytochemistry, pharmacological effects, and toxicity in recent decades. It is expected to provide a preliminary basis for future research on *E. ferox* and to provide a reference for further studies on the biological activities and clinical applications.

Firstly, *E. ferox* contains complex and diverse chemical constituents, including triterpenes, sterols, flavonoids, phenylpropanoids, essential oils, organic acids, and polysaccharides. So far, more than 100 compounds have been isolated and identified. However, although many chemical components have been elucidated, only a few of them have been validated for their biological activity. There is a lack of in-depth studies on the mechanisms of physiological activity of polysaccharides. The homosaccharide, structural information, monosaccharide composition, and content need to be further investigated.

Secondly, the anti-diabetes, gastrointestinal diseases, and even anti-cancer effects of *E. ferox* have been greatly explored. However, the understanding of its mechanisms and pathways of action remains ambiguous and is mostly based on its anti-oxidant effects. In addition, there are few studies that addressed the toxicities of *E. ferox*, and the pharmacokinetics and drug interactions of *E. ferox* in vivo remain unknown.

Further research and development are necessary for the following aspects. Firstly, it is important to continue isolating, purifying, and identifying chemical components, with emphasis on the biological activity and structure-activity relationships. Secondly, more in-depth studies should be conducted to determine the mechanisms of the physiological activity of polysaccharides present in *E. ferox*. This will shed more light on its biological activities and clinical applications. Thirdly, new techniques and methods such as molecular biology, cell biology, and histology should be combined to obtain intuitive evaluations via animal and clinical experiments and further explore intrinsic pharmacological mechanisms.

As a medicinal food ingredient with rich nutritional value and various health functions, *E. ferox* have good prospects for market development. The design of *E. ferox* special functional food and the utilization of its derived waste material might be the future directions.

## Figures and Tables

**Table 1:** The traditional uses of *E. ferox*.

Parts	Herbal Records	Dynasty/Country	Effects	Ref.
Seeds	Shen Nong’s Classic of the Materia Medica (Shén Nóng Bĕn Căo Jīng, 神农本草经)	Eastern Han Dynasty, AD 25–220, China	Eliminating dampness, easing backache and knee pain, tonifying and removing malignant diseases, benefiting the essence, strengthening the will, and making the ears and eyes wise.	[5]
	The Compendium of Materia Medica (Bĕn Căo Gāng Mù, 本草纲目)	Ming Dynasty, AD 1578, China	Quenching thirst and benefiting the kidney, treating urinary incontinence, spermatorrhea, and leucorrhea.	[28]
	The Song of Medicinal Properties and four hundred flavours (Yào Xìng Gē Kuò Sì Bǎi Wèi Bái Huà Jiě, 药性歌括四百味)	Ming Dynasty, AD 1581, China	Benefits the essence, relieving soreness of the waist and knees, and arresting seminal emission.	[29]
	Leigong Concocted Medicinal Annotation (Léi Gōng Páo Zhì Yào Xìng Jiě, 雷公炮制药性解)	Ming Dynasty, AD 1622, China	Tonifying the spleen and stomach, benefitting the essence, improving visual and auditory acuity, and amnesia.	[29]
	Essentials of Chinese Materia Medica (Běn Cǎo Bèi Yào, 本草备要)	Kangxi XXXIII, AD 1694, China	Strengthening the kidney and benefiting the essence, tonifying the spleen, and eliminating dampness. Treating diarrhea with turbidity and spermatorrhea.	[29]
	Chinese Pharmacopoeia	AD 2020, China	Benefiting the kidney and consolidating sperm, tonifying the spleen and inhibiting diarrhoea, eliminating dampness and arresting leucorrhea, improving spermatorrhea, enuresis and frequent urination, splenoasthenic diarrhea, and leucorrhea.	[9]
	Traditional Medical & Pharmaceutical Database	Japan	Improving metabolic arthritis, urinary incontinence, and leucorrhea, easing waist pain.	-
	Ayurveda and Unani system	India	Improving rheumatic and bile disorders, against dysmenorrhea and exerting spermatogenic properties.	[27]
Stems	The Compendium of Materia Medica (Bĕn Căo Gāng Mù, 本草纲目)	Ming Dynasty, AD 1578, China	Quenching irritability and thirst, eliminating asthenia-heat syndrome.	[28]
Leaves	The Compendium of Materia Medica (Bĕn Căo Gāng Mù, 本草纲目)	Ming Dynasty, AD 1578, China	Treating retained placenta and haematemesis.	[28]
Roots	The Compendium of Materia Medica (Bĕn Căo Gāng Mù, 本草纲目)	Ming Dynasty, AD 1578, China	Improving swollen testicles, and abdominal pain due to stagnation of vital energy.	[28]

**Table 3:** Pharmacological activities of *E. ferox*-derived extracts and phytoconstituents.

Activity	Compound/Extract	Plant Part	Animals/Cell Lines	Doses	Effects	Ref.
Antioxidation and anti-inflammation	Methanol extract	Seeds	DPPH scavenging assays V79-4 cell line RAW 264.7 cell line	For antioxidation, 0.8–100 μg/mL For antiinflammation, 100–400 μg/mL	DPPH radical scavenging activity (IC_50_ 22.95 ± 0.25 μg/mL or 5.6 μg/mL); iNOS, Cox-2, NO inhibition (300–400 μg/mL); Inhibition of lipid peroxidation (IC_50_ 20.5 μg/mL)	[19,20]
	Ethanol extract Compounds **40**, **42**–**46**	Seeds	DPPH scavenging assays ROS model: glucose-treated mesangial cells	For DPPH: 2–1000 μg/mL For ROS: 1, 10 μM	DPPH (SC50) of ethanol extract, compounds **40**–**43**, **45** were 103.1 μg/mL, 6.8, 10.4, 10.2, and 12.9 μM, respectively; Compounds **40**, **42**, **44**–**46** showed ROS inhibition at 10 μM	[39]
	Aqueous extracts	Seeds	DPPH and ABTS scavenging activity H_2_O_2_-induced human skin fibroblast oxidative stress	/	DPPH and ABTS scavenging; Increased expression of SOD, CAT, and GSH-Px	[21]
	Phenolic extracts	Seed shells	DPPH scavenging assays	0.1–1.0 mg/mL	DPPH scavenging activity is similar to vitamin C and Trolox at 1 mg/mL	[36]
	Phenolic extracts	Seed shells	DPPH and Hydroxyl scavenging assays D-galactose-induced aging Kunming mice	In vitro: 0.01–2 mg/mL In vivo: 100, 200, 400 mg/kg p.o. once daily for 32 days	Strong DPPH and Hydroxyl scavenging activity; Increases the SOD, CAT, and GSH-Px activities, and decreases MDA content in the liver and kidneys	[18]
	Anthocyanins extraction	Leaves	DPPH and ABTS scavenging activity	/	DPPH and ABTS scavenging IC_50_ were 74.00 ± 3.63 μg/mL and 5.77 ± 0.28 μg/mL, respectively	[26]
	Ethyl acetate, ethanol, or 50% ethanol extract	Seed shells	DPPH scavenging assays Lipid peroxidation	5–200 μg/mL	DPPH radical scavenging activity (IC_50_ 29.4 ± 1.34, 28.3 ± 1.21, 27.60 ± 1.02 μg/mL, respectively); Inhibition of lipid peroxidation (IC_50_ 43.86 ± 1.32, 30.44 ± 1.15, 36.42 ± 1.43, respectively)	[54]
	Cell wall polysaccharides	Petioles and pedicels	DPPH and ABTS scavenging activity H_2_O_2_-induced injury on HUVEC and VSMC	For DPPH and ABTS: up to 6.5 mg/mL For cell model: 60 and 200 μg/mL	Around 80% DPPH scavenging activity at 3.25 mg/mL, and 100% ABTS scavenging activity at 1.625 mg/mL; Reduced MDA levels, and increased T-AOC, SOD, and CAT activities in H_2_O_2_-injured VSMC, and HUVEC cells.	[55]
	Essential Oil	Seeds	DPPH and ABTS scavenging activity	0.5, 1, 2, 4, and 8 μg/mL	DPPH and ABTS scavenging IC_50_ were 6.27 ± 0.31 and 2.19 ± 0.61 μg/mL, respectively	[46]
Antidiabetic and hypoglycemic effects	70% ethanol extract	Seeds	Streptozotocin-induced diabetic Wistar rats	100, 200, 300, 400 mg/kg, p.o. for 45 days	Significantly decreased the blood glucose level, increased plasma insulin level, restored hepatic gluconeogenic enzymes activities; increased activities of SOD, CAT, GPx, and GSH	[15]
	70% methanol extract	Germinated seeds	Streptozotocin-induced diabetic ICR mice	100, 200, 400 mg/kg, p.o. for 4 weeks	Improved hyperglycemia, abnormal lipid metabolism, and renal tissue lesions; Decreased kidney microalbuminuria, blood urea nitrogen, serum creatinine, MDA, and GSH; Increased activity of CAT, SOD, serum total antioxidant capacity; Regulating the Keap1/Nrf2/HO-1 and AMPK/mTOR pathways.	[56]
	Polysaccharide (EFSP-1)	Seeds	Dexamethasone-induced HepG2 and 3T3-L1 preadipocyte cells	Incubation with 25, 100, 400 μg/mL EFSP-1 for 24 h	Increasing glucose consumption by up-regulating the expression of GLUT-4 via activating the PI3K/Akt signal pathway in insulin resistance cells	[32]
	2β-hydroxybetulinic acid 3β-caprylate (HBAC)	Seeds	Streptozotocin-induced diabetic Wistar rats	20, 40, 60 mg/kg, p.o. for 45 days	Exhibited free radical scavenging property, pancreas, and hepatoprotective effect; Stimulating insulin release; Improved the glycemic control and lipid profile	[44]
	2β-hydroxybetulinic acid 3β-oleiate (HBAO)	Seeds	Streptozotocin-induced diabetic Wistar rats	20, 40, 60 mg/kg, p.o. for 45 days	Alleviating glycemic homeostasis and oxidative stress, normalized plasma glucose, glycosylated hemoglobin (HbA1c), hepatic gluconeogenic enzymes, plasma insulin, ameliorating pancreatic β-cell, hepatic and renal histology and β-cell functions, improving dyslipidemia and antioxidant enzymes	[45]
	Ethanol extract	Seed shells	α-amylase and α-glucosidase	20–100 μg/mL	The inhibitory effects of *E. ferox* seed shell extract (EFSSE) on α-amylase and α-glucosidase in terms of IC_50_ were 62.95 and 52.06 μg/mL, respectively.	[57]
	Triterpenoid-rich 75% ethanol extracts	Seed shells	Streptozotocin-induced diabetic mice	200, 400, 600 mg/kg, p.o. for 4 weeks	Regulating glucose metabolism and body weight; Decreased cholesterol, LDL, and triglycerides levels, and increased HDL	[53]
	Triterpenoid-rich 75% ethanol extracts	Seed shells	Streptozotocin-induced diabetic Kunming mice	200, 300, 400, 500 ± 2 mg/L in drinking water for 4 weeks	Restored glucose metabolism and body weight; Recovered Islet morphology; Reduced PTP1B protein and increased insulin receptor IRS-1 protein	[58]
	Network pharmacology method	/	The TCMSP, SymMap V2, CTD, DisGeNET, and GeneCards databases were searched for ES components, targets, and DKD targets	/	The main components are oleic acid and vitamin E, targeting the proteins PPARA, LPL, FABP1, and MAPK1 to regulate TNF, apoptosis, and MAPK.	[59]
	Polysaccharides	Petioles and pedicels	Alloxan-induced hyperglycemic ICR mice	100, 200, 400 mg/kg, p.o. for 28 days	High doses of EFPP reverse alloxan-induced body weight loss, reduce blood glucose level, enhance serum insulin level, improve oral glucose tolerance, increase hepatic glycogen content and GCK activity; increase SOD, CAT, and GSH-Px activities and decrease MDA contents in liver and kidney	[60]
Hepatoprotective and cardioprotective activities	Ethanol extract	Seed shells	High-fat diet-induced ICR mice	15 and 30 mg/kg, p.o. for 28 days	Reduced body weight, lipids deposition in the liver and blood lipids, decreased MDA content, and increased SOD activity; IRs-1 activation and CYP2E1 inhibition	[13]
	Ethanol extract	Seeds	Ischemia and reperfusion in vitro model; Chronic ischemic reperfusion injury in vivo model	25, 125, or 250 μg/mL for in vitro; 50 and 500 mg/kg, p.o. for 21 days	Improved post-ischemic ventricular function and reduced myocardial infarct size; increased expression of TRP32 and thioredoxin proteins; ROS scavenging activities	[14]
Anticancer	Ethanol extract	Seeds	In vitro: A549 Human Caucasian Lung Carcinoma cancer cells In vivo: Balb/c nu/nu mice	In vitro: 50–150 μg/mL In vivo: 100 mg/kg/day for 28 days	In vitro: promoting A549 apoptosis via inhibition of the Akt protein and activation of the p53 protein; In vivo: activating p53 and suppressing the tumor growth	[16]
	Ethanol extract	Seed shells	Human Gastric Cancer SGC7901 cells and Human Hepatoma HepG2 cells	50–800 μg/mL	Inhibitory effect on the proliferation of SGC7901 cells and HepG2 cells were 92.63% and 72.40%, respectively	[61]
	Ethyl acetate fraction	Seeds	Melan-a cells	3–30 μg/mL	Inhibition of cellular tyrosinase and melanin synthesis	[23]
	Resorcinol	Seeds	B16F10 melanoma cells	/	Inhibition of melanin synthesis in B16F10 melanoma cells with an IC50 492.8 µM	[37]
Cytotoxicity	Ferocerebrosides A and B	Seeds	Brine shrimp lethality bioassay	62.5, 125, 250, 500, and 1000 μg/mL for 24 h	Ferocerebrosides A and B showed marginal toxicity against brine shrimp with LC50 values of 0.17 and 0.20 mM, respectively	[42]
	Polysaccharide fraction (EFSP-1)	Seeds	3T3-L1 preadipocyte cells and HepG2 cells	25, 50, 100, 200, 400 μg/mL for 48 h	No obvious influence on cells at 100–400 μg/mL	[32]
	Hexane, diethyl ether, ethyl acetate extract	Seeds	Glutamate-induced cytotoxicity in hybridoma N18RE-105 cells	10 μg/mL for 24 h	Dose-dependent protection against neuronal cell death induced by 20 mM glutamate	[62]
Anti-fatigue	Phenolics extract	Seed shells	Exhaustive swimming test	100, 200, 400 mg/kg p.o. once daily for 32 days	The average exhaustive swimming time was obviously prolonged in all three doses	[18]
Anti-depressant	Petroleum ether fraction	Seeds	Chronically unpredictable mild stress (CUMS) mouse model	0.1, 0.15 g/kg p.o. once daily for 14 days	Upregulation of AMPK and ULK1, attenuated depressive behavior via AMPK-ULK1 pathway-mediated autophagy	[22]

## Data Availability

Data sharing not applicable to this article as no datasets were generated or analysed during the current study.

## Sample Availability

Not available.
